# Personal and Professional Mitigation Behavioral Intentions of Agricultural Experts to Address Climate Change

**DOI:** 10.1007/s00267-023-01815-y

**Published:** 2023-04-03

**Authors:** Tahereh Zobeidi, Masoud Yazdanpanah, Laura A. Warner, Alexa Lamm, Katharina Löhr, Stefan Sieber

**Affiliations:** 1 Cooperation and Transformative Group, International Institute for Applied Systems Analysis (IIASA), Laxenburg, Austria; 2 Department of Agricultural Extension and Education, Agricultural Sciences and Natural Resources University of Khuzestan, Mollasani, Iran; 3 Department of Agricultural Education and Communication, Institute of Food & Agricultural Sciences, University of Florida, Gainesville, FL USA; 4 Department of Agricultural Leadership, Education and Communication, College of Agricultural and Environmental Sciences, University of Georgia, Athens, GA USA; 5 Leibniz Centre for Agricultural Landscape Research (ZALF), Muncheberg, Germany; 6 Urban Plant Ecophysiology, Humboldt-Universitat zu Berlin, Berlin, Germany; 7 Resource Economics, Humboldt-Universitat zu Berlin, Berlin, Germany

**Keywords:** Mitigation, Risk salience, Intention to act, Hypothetical distance, Climate change

## Abstract

Mitigation activities, whether at the personal level relating to lifestyle or on the professional level, especially in the agriculture sector, are widely encouraged by scientists and policymakers. This research empirically analyses the association between agricultural experts’ perceptions about climate change and their intention to implement climate change mitigation. Based on survey data, individuals’ reported intention to implement personal and professional mitigation behavior is explained using a conceptual model. The structural equation modeling results suggest that the new ecological paradigm (NEP), institutional trust, and risk salience indirectly influence climate change mitigation intentions. The findings indicate that risk perception, personal efficacy, responsibility, belief in climate change occurring, and low psychological distance trigger a significantly greater intention to support personal and professional mitigation behaviors. However, the research framework is much stronger at predicting the intention to mitigate climate change in professional affairs compared to personal activities. The findings suggest that hypothetical distance factors only have a moderating effect on the relationship between higher climate change environmental values, institutional trust, risk salience, and mitigation intention. This paper analytically explores the regulating role of risk perception, hypothetical distance, personal efficacy, and responsibility between institutional trust, risk salience, and the NEP as independent concepts and intention to personal and professional mitigation behaviors as dependent variables. The findings of the study have important implications for encouraging personal and professional mitigation behaviors.

## Introduction

Climate change is one of the most challenging social issues of the current era. As of 2023, anthropogenic activities have pushed global temperatures up by about 1.0 °C compared to pre-industrial levels. If current emission rates continue, this figure is likely to reach 1.5 °C between 2030 and 2052 (Fawzy et al. 2020) and 2 °C before 2100 (Malhi et al. 2021). Statistics show that by 2030 and 2050, developing countries will be responsible for up to 64% and 76% of global greenhouse gas emissions, respectively (Sohoo et al. (2020)). Studies using global data from 1970 onwards show that global warming significantly affects many physical and biological systems (Parry 2007). Climate change due to increasing resource scarcity, escalating natural disasters, and rising sea levels can lead to social instability and conflict (Brown et al. 2013). In particular, natural resource-dependent communities tend to be impacted by climate change more severely and, consequently, increasingly struggle to maintain sustainable livelihoods, including ecological and human well-being (Suckall et al. 2014). Climate change threatens the agricultural sector and resource dependent communities through increased soil erosion, reduced soil quality, reduced agricultural production, and decreased food security (Gezie (2019); Loboguerrero et al. 2019; Karimi and Ataei 2022; Aliabadi et al. 2022). In this sense, countries that rely strongly on agricultural production, including most developing countries, are more vulnerable to climate change (Bryan et al. 2009).

Agriculture, at the same time, also contributes to climate change. For example, it is a source of three greenhouse gases (GHGs): nitrous oxide (N2O), methane (CH4), and carbon dioxide (CO2) (de Oliveira et al. (2019)). Significant GHGs are released through crop and soil management, manure management, and methane production during animal digestion; the latter a process called intestinal fermentation (Massey and Ulmer 2010). The use of chemical nitrogen fertilizers, important inputs in modern agricultural systems, leads to the production of direct and indirect N2O (Elrys et al. 2020), which is a powerful factor in global warming. Over a period of 100 years, this gas is 298 times more effective at heating the atmosphere than CO2 (Gu et al. (2017)). In addition, CO2 is released to a large extent from microbial decay or incineration of plant wastes and soil organic matter (Pradisty et al. 2021; Praveen and Sharma 2019). Arbuckle et al. (2013) indicate that production systems are a significant emitter of CO2 and estimate that agriculture produces between 10 and 15% of the world’s human GHGs emissions. Therefore, controlling and reducing GHGs from the agricultural sector is an essential measure and one of the most important mechanisms to prevent the adverse effects of climate change (Venkateswarlu and Shanker 2009). Efforts to reduce GHGs emissions, like climate change mitigation strategies, are widely endorsed by scientists and policymakers, with positive effects linked to both human and natural systems (Schuldt et al. 2018).

Climate change calls for a range of mitigation measures, from measures implemented by law to change technologies, to changes that citizens voluntarily undertake in their daily behavior (Semenza et al. 2008; Chen 2020a). The word “mitigation” refers to reduction of GHGs, with mitigation activities (i.e., reducing the causes of climate change) resulting in reduced GHGs emissions from the source or by replacing and conserving energy, improving sedimentation carbon, etc. (Dhillon and von Wuehlisch 2013; Honegger et al. 2021; Wang et al. 2021). Since most scientists consider the phenomenon of global warming to be characterized by human activities, they want people to participate in measures that reduce the emissions of heat trapping gases, thus reducing the negative effects of global warming (Kahan (2015)).

Agriculture studies show that even small changes in farm practices (e.g., decreasing nitrogen manure use) (Hamid et al. (2021)) can greatly reduce GHGs emissions (Sanz-Cobena et al. (2017)). Thus, farmers’ role in carrying out mitigation activities in farming is important and vital. However, studies in Iran reveal that farmers’ awareness about climate change and mitigation is still not adequate (Yazdanpanah et al. 2022b). Because of this, agricultural experts can play a fundamental role for encouraging and facilitating these initiatives. Meanwhile, agricultural advisors play an important role and can potentially have a great impact on farmers’ climate change mitigation behavior. Agricultural experts can play an important role in finding regionally suitable solutions that reduce GHG emissions from agricultural and livestock activities as well as in providing them to farmers. Agricultural experts can also play an important role in influencing the acceptance of agricultural innovations by farmers (Wheeler 2008) or they can influence the attitudes and behaviors of others (Ghasemi et al. 2013). Indeed, agricultural advisors are an acknowledged trusted source of information for farmers, so understanding their beliefs and conveyed messages are important for better understanding farmers’ decisions regarding climate change (Chatrchyan et al. 2017).

Extension services use agricultural experts to prepare farmers by providing training on best farming practices, thus increasing the level of acceptance of new technologies. Empowering farmers to deal with different forms of climate change risks is very important and, to achieve this, special attention should be paid to teaching options that increase their capacity building. A number of studies investigate the importance of agricultural professionals in raising awareness and encouraging and educating farmers (and the public) about initiatives like mitigation behavior through teaching and extension work (Ghasemi et al. 2013; Bakhtiyari et al. 2017). Agricultural professionals can act as gatekeepers (Bakhtiyari et al. 2017), either facilitating or hampering the adoption of an initiative (Yaghoubi et al. 2019). In the same vein, Karppinen (2005) states that experts are amongst the most important supporters, consultants, and instructors that growers rely on as a trusted source of evidence. Gautam et al. (2013) point out that insights of agricultural agents are key because they are formed by their experience in the sector and local conditions in which they operate, particularly in remote and rural areas.

To better plan and implement new policies and build capacity, it is crucial to understand those factors driving behavior. Researchers find that external and internal factors influence environmental behavior, including demographic factors and psychological factors such as values, beliefs, perceptions, attitudes, and intentions (Brown et al. 2019). Changing people’s perceptions of climate change and increasing their participation in reducing GHG is essential for a successful transition to a low-carbon economy (Capstick et al. 2015; Wibeck 2014). Perception development is an active process based on what exists externally as well as the internalized experiences, desires, needs, preferences, and dislikes of individuals themselves (Euriga et al. 2021). In the context of climate change, an individual’s response may be largely determined by their perceptions of the problem itself rather than their attitudes toward specific behaviors (Hu and Chen 2016). Semenza et al. (2008) point out that voluntary reduction of energy consumption by individuals – conditioned on their awareness and concern about climate change, their willingness to act, and their ability to change – is an important factor in counteracting climate change. Public support for, or opposition to, climate policies (e.g., treaties, regulations, taxes, subsidies) is largely influenced by the public perception of those risks and harms of exposure to global climate change (Leiserowitz 2006). Committees of the US National Research Council (National Research Council (1992)), for example, the Committee on the Human Dimension of Global Change, have identified public perceptions of global phenomena, such as climate change, as a critical factor in both environmental problems and possible solutions (Weber 2010).

There are few studies examining the determinants of climate change mitigation intentions in agriculture. For example, Zhang et al. (2020) and Niles et al. (2016), examine predictors of intention of mitigation using the theory of planned behavior (TPB) and value-belief-norm theory (VBN), while Chen (2020a, 2020b) did so using protection motivation theory and construal level theory (CLT). To enhance knowledge on the effect of psychological factors on mitigative intention, this study uses a conceptual theory based on beliefs (hypothetical distance) and risk perception to predict the intention of agricultural experts using the case of Iran. To the best of our knowledge, despite the importance of both the beliefs and perceptions of agricultural experts in terms of education and orientation to agricultural education for mitigation, no such study has been undertaken. Further, those studies that exist on mitigation behavior (Ferguson and Branscombe 2010; Spence et al. 2011; Ambusaidi et al. 2012; Sinatra et al. 2012; Broomell et al. 2015; Hu and Chen 2016; Niles et al. 2016) do not study it in the context of Iran. Therefore, this study presents novelty in several ways. First, from a scientific perspective, this study attempts to provide a conceptual framework for mitigation given foundational theories used in previous studies. In addition, the constructs considered by this study have a direct relationship with climate change and, for this reason, they seem more appropriate than theoretical frameworks. However, some constructs, like hypothetical distance and risk perception, are derived from well-known theories. The second novelty is that this study uses a research sample from Iran, a developing country whose farmers are generally low in literacy. This study seeks to identify which psychological factors affect the intentions of agricultural experts to engage in personal and professional mitigation behaviors in agriculture to reduce climate change.

### Conceptual Framework

In this study, we assume that belief in the existence of climate change is pivotal in addressing the tensions between the abstractness of global climate change and the concrete intentions to mitigate climate change locally. To develop our conceptual framework on the intensions of experts to mitigate climate change, we examine the effect of hypothetical distance and risk perception on the intention to engage in mitigation behaviors. Hypothetical distance, one aspect of psychological distance, refers to uncertainty regarding occurrence of a phenomena (see, Spence et al. 2012; McDonald et al. 2015; Maiella et al. 2020). Hypothetical distance is related to the perceived probability (likelihood or unlikelihood) of occurrence or non-occurrence of an event (McDonald et al. 2015). The shorter the hypothetical distance, the more people believe in the occurrence of climate change. Uncertainty about the occurrence of climate change often leads to people not fully understanding the various associated predictions and, therefore, inaccurately analyzing the probability of its occurrence (Maiella et al. 2020). For example, people who are skeptical about climate changes are less likely to adapt their behavior (Spence et al. 2012). Researchers believe that perceived or actual uncertainty reduces the number of times individuals engage in pro-environmental behavior (Gifford (2011); Aitken et al. 2011). Without a belief that climate change is happening, people pay little attention to actions required to address it. Therefore, reduced hypothetical distance and greater belief in the occurrence of climate change will increase people’s willingness to support mitigation measures. Many people do not always behave in a sustainable way, which is said to be partly because they perceive climate change as a distant psychological issue (Spence et al. 2012). Some studies argue that reducing hypothetical distance and relating to it can increase the likelihood of behavior change (McDonald et al. 2015; Schuldt et al. 2018).

Past research demonstrates that perceived risk (judging of the severity and urgency of the problem of climate change) is the strongest predictor of actions. Those who acknowledge that the risks of climate change are high are more likely to act (Kollmuss and Agyeman 2002; Aitken et al. 2011). Arbuckle et al. (2013) show that farmers who are concerned about the effects of climate change on agriculture have more supportive mitigation measures. Feeling worried, or perceiving danger, is one of the most important factors in determining whether people engage in professional environmental behavior (Kollmuss and Agyeman 2002).

However, climate change is considered a “dead” hazard among similar natural processes, like temperature changes and climate fluctuations, with little salience as a high-risk issue because it is not directly experienced (Whitmarsh 2008). While it is difficult to judge climate change as an abstract concept based on personal experience (Weber 2010), risk salience is a factor influencing the perception of climate change, which comprises two components of close proximity to risk and related previous experience (Carlton and Jacobson 2013). Research emphasizes that experience influences risk perception. Previous experience may affect the perception of danger by engaging people cognitively. People who have suffered from an environmental catastrophe are more likely to remember and relate their perceived risks when considering environmental hazards. Past studies also predict that personal experiences or relationships with local climate and extreme climatic events cause climate change to move from being abstract to being a familiar, real, and immediate concept (Akerlof et al. 2012). Personal or direct experience may be a factor that influences the intention to implement mitigation behaviors (Ogunbode et al. 2019; Capstick et al. 2015). Existing research shows that environmental views and perceptions of climate change can be related to people’s physical surroundings and their experiences (Spence et al. 2011). For example, Drummond and Palmer (2014) show that belief in global warming increases when physical heat is experienced by being in a heated room.

Feeling personal efficacy and responsibility are other factors influencing risk perception, with people who feel less effective and responsible for climate change having less concern or risk perception (Kellstedt et al. 2008). Personal efficacy is a central concept used in health studies. Heath and Gifford (2006) also predict the effectiveness of responses in general and/or the belief that their efforts to reduce global warming will make a difference are predictors of intention. Stoutenborough and Vedlitz (2014) show that those who have most perceived efficacy are motivated to better understand climate change. Whitmarsh (2009) also believes that behavioral intentions to address climate change are influenced by perceived responsibility to cause and respond to climate change.

Scientists argue that trust is an important factor determining public perceptions of climate change and, consequently, support of mitigation efforts (Hmielowski et al. (2014)). Trust is defined as an expectation that empowers and motivates the trustee to behave in a way that is valued by the trustor (Hmielowski et al. (2014)). Cologna and Siegrist (2020) believe that individuals who make choices in circumstances of uncertainty and poor knowledge, like climate change, tend to rely on trusted institutions for guidance, with the level of trust determined by public acceptance. Chryssochoidis et al. (2009) point out that institutional trust is flexible, typically shaped by socio-cultural factors and value systems.

The New Environmental or Ecological Paradigm (NEP; Dunlap 2008) is broadly recognized as a reliable multiple‐item scale to capture environmental attitudes (Yu et al. 2019) or environmental values (Ziegler (2021)). NEP consider a set of basic beliefs about humankind’s association with environment, containing the concept that current civilizations have harmed the stability of nature, restricted growth, and the necessity to shed an anthropocentric orientation toward the environment. This set of beliefs is grounded in the notion that these beliefs are more central than attitudes toward precise issues, like support for contamination regulator (Amburgey and Thoman 2012).

**Fig. 1:**
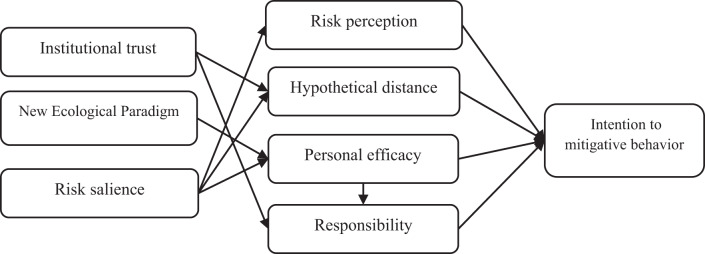
Conceptual framework of personal and professional mitigation intention

NEP assumes that an environmental behavior is the result of individual environmental worldviews, reflecting people’s beliefs about humanity’s ability to disturb nature’s balance, the existence of growth restrictions on human societies, and human rights to rule over nature (Chen 2020a). NEP, according to Dunlap (2008), is a standard instrument in social and behavioral disciplines that is increasingly used as an indicator for environmental values, concern, awareness, or attitudes in economics (Ziegler (2021)). Environmental attitudes and values reflect having a good understanding of a set of beliefs, interests, or laws that influence environmental protection behavior (Rodríguez-Barreiro et al. 2013). Various studies show that environmental values (NEP) can influence many factors. For example, Wang et al. (2022) and Sarrasin et al. (2022) find that pro-environmental values can influence perceived behavioral control or self-efficacy. Stoutenborough and Vedlitz (2014) and Kellstedt et al. (2008) find that those with higher ecological values are likely to try and understand climate change because of their environmental concerns (see Fig. 1).

## Methodology

### Survey

The purpose of this study is to identify factors affecting the intention to implement personal and professional GHGs emission mitigation behaviors by agricultural experts. Personal mitigation behaviors include walking, riding bicycles, taking public transportation, choosing eco-friendly products, avoiding the purchase of out-of-season food, reusing and repairing items instead of throwing them away, paying more taxes to combat climate change, as well as saving on paper and napkins. Professional mitigation behaviors in an agricultural context include increasing organic agriculture, using renewable energy, using less polluting and more energy efficient machinery, as well as zero tillage management (Kreft et al. 2021).

This research was conducted as a non-experimental cross-sectional survey to test the hypotheses of the conceptual framework. Data collection was conducted using face-to-face questionnaires with randomly selected agricultural professionals (*n* = 320) in Khuzestan province, Iran, from the two majors agricultural organization (Agricultural-Jihad centers) in 2020.

The distribution of socio-economic characteristics data of these experts shows that more than half the sample are male: 178 (55.6%) males; 142 (44.4%) females. The average age is 35.53 (SD = 8.10) ranging from 22 to 70. Average work experience is 9.12 (SD = 7.56) years, ranging from 0 to 38 years. A significant percentage of experts have a Bachelor’s degree 163 (50.9%). After that, 29.7% (95) have a Master’s degree and 12.2% (39) a Doctorate. Two people (6.0%) have an Associate’s degree, while 13 (4.1%) have a diploma and 8 (2.5%) did not answer this question.

The respondents were asked to respond to statements using a 5-point Likert scale, with 1 = strongly disagree; 2 = disagree; 3 = neutral; 4 = agree; and 5 = strongly agree. The survey items are adapted from several studies, with the items, sources, and descriptive statistics shown in Table 1. The reliability and validity of the survey data were tested using SPSS 23.0. The results show that Cronbach’s alpha values for all constructs are higher than 7.0, thus indicating good internal consistency (Nunnally 1978).

**Table 1:** Sources, items and descriptive statistics of survey items

Constructs	Items	Factor loading	Cronbach’s alpha	Means	S.D.
Institutional trust Stoutenborough and Vedlitz 2014	Government agencies provide accurate and reliable information on climate change.	0.84	0.85	3.03	1.15
	Government agencies provide timely information on climate change.	0.88		2.82	1.27
Risk salience Carlton and Jacobson 2013	I know people who have problems due to extreme weather.	0.75	0.83	3.64	1.19
	I have never seen a crop or farm that has a problem due to climate change.	0.82		3.61	1.18
	I see that the farms in this area are damaged due to climate change.	0.80		3.38	1.22
NEP Dunlap et al. 2000	I do not think climate change has increased, only these days the media reports on it more.	0.71	0.76	3.86	1.13
	What is called climate change is just a natural fluctuation in the earth’s temperature.	0.63		3.72	1.17
	Information about climate change and global warming does not apply to me.	0.68		4.21	1.14
	I feel a moral duty to do something to combat climate change.	0.58		3.89	1.14
Personal Efficacy Kellstedt et al. 2008 Yazdanpanah et al. 2022a	I can easily train farmers to produce less GHG.	0.69	0.83	3.28	1.06
	If you want, I can easily educate farmers about the effects of climate change.	0.76		3.19	1.08
	In my daily life, I can easily do things that reduce GHG emissions.	0.76		3.32	1.14
	I can easily train farmers to do things that emit less GHG.	0.76		3.38	1.18
Responsibility Kellstedt et al. 2008	I feel responsible for the contribution of agriculture to global warming.	0.78	0.83	3.70	1.08
	We humans are responsible for climate change.	0.78		3.82	1.09
	Tackling climate change is not only the responsibility of government and industry but also individuals like me.	0.74		3.92	1.07
	Every citizen is responsible for reducing GHG emissions.	0.72		3.72	1.20
Risk perception/public concern Leiserowitz 2006; Kellstedt et al. 2008	In the next 25 years, global warming and climate change will have significant negative effects on human health.	0.64	0.83	4.05	0.88
	In the next 25 years, global warming and climate change will have significant negative effects on my economic and financial situation.	0.60		3.67	0.97
	In the next 25 years, global warming and climate change will have significant negative effects on the health of the Iranian people.	0.75		4.09	0.88
	In the next 25 years, global warming and climate change will have significant negative effects on Iran’s economic development.	0.70		4.01	0.85
Belief in happening/ low Hypothetical distance Spence et al. 2012; Arbuckle et al. 2013	Variability in precipitation is more than in previous years.	0.78	0.87	4.00	1.12
	Winters are not as cold as in previous years.	0.78		3.97	1.07
	The current summers are warmer than the previous years.	0.73		4.07	1.04
	Compared to previous years, annual precipitation has decreased.	0.85		4.06	1.05
Personal mitigation intention Chen 2020a	Buy eco-friendly products.	0.59	0.85	4.38	1.27
	Do not buy food out of season.	0.66		4.37	1.36
	Walk, ride bicycles, or use public transportation.	0.64		4.42	1.24
	Reuse and repair items instead of throwing them away.	0.70		4.65	1.30
	Pay more taxes to combat climate change.	0.80		4.71	1.19
	Save on paper and napkins.	0.67		4.63	1.12
Professional mitigation intention Fawzy et al. 2020 Zhang et al. 2020	Because of my commitment to future generations, I intent to teach ways to reduce GHG emissions from livestock.	0.73	0.85	3.96	1.00
	I intend to teach decarbonization technologies, such as the use of renewable energy in agriculture.	0.75		3.45	1.25
	I intend to teach using no-till and direct seeding methods.	0.72		3.27	1.08

### Data Analysis

Structural Equation Modeling (SEM) is a regression-based technique that is frequently applied to validate hypothetical or theoretical models (Ho et al. 2016). SEM can calculate measurement error and can simultaneously estimate model path coefficients (Fan et al. 2016). Therefore, SEM analysis is used to evaluate the validity of the measurement model and the explanatory power of the structural model in predicting the intention of agricultural experts to implement personal and professional mitigation behaviors.

The two stages of SEM include confirmatory factor analysis (CFA) to appraise the suitability of the measurement model and then modeling of structural equations (Anderson and Gerbing 1988). CFA was performed using AMOS 23 software. CFA is validated using indicators such as Goodness of Fit Index (GFI), Root Mean Square (RMS), Comparative Fit Index (CFI), and the Normed Fit Index (NFI). A model is considered as acceptable if the NFI exceeds the threshold of 0.90, GFI is greater than 0.90, CFI is more than 0.90, and RMSEA is less than 0.08 (Hair et al. 2010). CFI was also used to assess the superiority and suitability of the measurement model by investigative convergent validity and discriminant validity.

To confirm the reliability of the research constructs, two indices including the Composite reliability (CR) and Cronbach’s Alpha were used. The CR is the consistent reliability of all measurement variables and its index meaning is like Cronbach’s alpha, representing the degree of internal consistency of the latent constructs. Cronbach’s alpha and CR values should all be more than 0.7. The results satisfied these requirements with Cronbach’s alpha values ranging from 0.76 to 0.87 and CR values ranging from 0.74 to 0.77, indicating high internal consistency and confirming good reliability of scales in this study (Table 2).

**Table 2:** Correlation between constructs and convergent validity

	Institutional trust	Risk salience	NEP	Risk perception	Personal Efficacy	Responsibility	Belief in happening	Personal mitigation	Professional mitigation
Institutional trust	1								
Risk salience	0.360**	1							
NEP	0.33**	0.64**	1						
Risk perception	0.16**	0.30**	0.17**	1					
Personal Efficacy	0.40**	0.51**	0.49**	0.17**	1				
Responsibility	0.19**	0.52**	0.56**	0.14**	0.55**	1			
Belief in happening	0.25**	0.67**	0.66**	0.24**	0.38**	0.55**	1		
Personal mitigation	0.25**	0.47**	0.53**	0.06	0.54**	0.56**	0.55**	1	
Professional mitigation	0.31**	0.56**	0.56**	0.09	0.66**	0.72**	0.55**	0.61**	1
CR	0.851	0.833	0.746	0.769	0.831	0.842	0.866	0.777	0.836
AVE	0.74	0.625	0.425	0.456	0.552	0.571	0.618	0.538	0.462

**p < 0.01.

To confirm convergent validity, the AVE of each construct should be higher than 0.5 (Fornell and Larcker 1981). As shown in Table 2, the current AVE values ranged from 0.42 to 0.74, thus showing good convergent validity of this study (Yu et al. 2019). The AVE values of all constructs except risk perception, NEP, and intention to professional mitigation, were lower than the threshold of 0.5 and the CR of all constructs was 0.7 (Table 2). Fornell and Larcker (1981) note that if the CR of a construct is higher than 0.7 then AVE values between 0.4 and 0.5 can be considered acceptable (Table 2).

The results of CFA show that all standardized factor loadings were greater than 0.6 and significant at the critical level of 0.01, indicating good discriminant validity. Meanwhile, the estimation of the parameters between the measured items and the conforming structures is statistically significant at the level of 0.01, which shows that each measured item has a strong ability to explain its corresponding latent construct.

Based on Fornell and Larcker (1981), discriminant validity was measured by using paired analysis of correlation coefficients. Comparison of the squared root of AVE and paired variable coefficients demonstrates that the squared root of AVE is higher than the correlation coefficients, representing the existence of discriminant validity. The consequences show that the square of the AVE of the latent variables studied is higher than the correlation of that latent with all other variables; therefore, the research tool has good discriminant validity.

In a structural model, after confirmation of measurement model, the degree of direct and indirect impact between the variables is examined. Here, the findings are presented in two separate structural models, including model 1 to formulate and test the hypotheses in a model that determines personal mitigation behaviors and model 2 for professional mitigation behaviors.

## Results

### Correlation between Constructs of the Model

The results show that all latent model constructs, except risk perception, have a significant correlation with the intention to implement personal and professional mitigation behaviors.

### Verification of Measurement Model

The goodness-of-fit indices of the two CFA model are as follows: model 1: chi-square value is 649.733 (df = 395), *p* = 0.000, relative chi-sq = 1.645, GFI = 0.885, CFI = 0.948, IFI = 0.949, RMSEA = 0.045, SRMR = 0.0467.

Model 2: chi-square value is 598.083 (df = 312), *p* = 0.000, relative chi-sq = 1.917, GFI = 0.883, CFI = 0.938, IFI = 0.939, RMSEA = 0.054, SRMR = 0.0493. These results indicate that the conceptual models of personal and professional mitigation behaviors fit the practical data with acceptable validity.

### Structural Models of Intention to Personal and Professional Mitigation Behaviors

The goodness-of-fit indices of the two structural models are as follows: model 1: chi-square value is 750.771 (df = 410), *p* = 0.000, relative chi-sq = 1.827, GFI = 0.869, CFI = 0.931, IFI = 0.931, RMSEA = 0.051, SRMR = 0.0513.

Model 2: chi-square value is 703.398 (df = 327), *p* = 0.000, relative chi-sq = 2.151, GFI = 0.860, CFI = 0.924, IFI = 0.919, RMSEA = 0.060, SRMR = 0.0539. These results indicated that the conceptual models of personal and professional mitigation behaviors fit the practical data with acceptable validity.

Based on the findings, conceptual model 1 predicts 68% of the intention to implement personal mitigation behaviors. As shown in Table 3, institutional trust (β = 0.206, *t* = 3.637, *p* < 0.0001), and risk salience (β = 0.929, *t* = 11.998, *p* < 0.0001) predict 74% of hypothetical distance or belief in the existence of climate change. The lower the trust in the government, the greater the confidence and belief in climate change. The greater risk salience, the more people believe in climate change. Model 1 predicts 13%, 46%, and 60% of the variance changes in risk perception, personal efficacy, and responsibility, respectively.

**Table 3:** Estimates of the structural models

Hypothesis			Unstandardized Regression Weights	SE	Standardized Regression Weights	C.R	sig	Results
Model 1								
Institutional trust	→	Hypothetical distance	0.189	0.052	0.206	3.637	*	Supported
Risk salience	→	Hypothetical distance	0.869	0.072	0.929	11.998	*	Supported
Institutional trust	→	Personal Efficacy	0.207	0.057	0.235	3.676	*	Supported
Risk salience	→	Risk perception	0.226	0.044	0.358	5.152	*	Supported
Risk salience	→	Personal Efficacy	0.068	0.185	0.075	0.367	0.714	Rejected
NEP	→	Personal Efficacy	0.608	0.276	0.475	2.203	0.028	Supported
Personal Efficacy	→	Responsibility	0.434	0.063	0.457	6.921	*	Supported
Hypothetical distance	→	Responsibility	0.406	0.058	0.445	6.964	*	Supported
Personal Efficacy	→	Personal mitigation	0.300	0.066	0.345	4.518	*	Supported
Responsibility	→	Personal mitigation	0.235	0.083	0.256	2.837	0.005	Supported
Risk perception	→	Personal mitigation	0.215	0.068	0.173	3.155	0.002	Supported
Hypothetical distance (Belief in happening)	→	Personal mitigation	0.343	0.064	0.411	5.374	*	Supported
Model 2								
Institutional trust	→	Hypothetical distance	0.182	0.052	0.198	3.521	*	Supported
Risk salience	→	Hypothetical distance	0.860	0.072	0.923	11.972	*	Supported
Institutional trust	→	Personal Efficacy	0.188	0.055	0.218	3.421	*	Supported
Risk salience	→	Risk perception	0.224	0.044	0.357	5.136	*	Supported
Risk salience	→	Personal Efficacy	0.081	0.171	0.093	0.475	0.635	Rejected
NEP	→	Personal Efficacy	0.613	0.258	0.491	2.374	0.018	Supported
Personal Efficacy	→	Responsibility	0.451	0.065	0.474	6.924	*	Supported
Hypothetical distance	→	Responsibility	0.383	0.058	0.430	6.620	*	Supported
Personal Efficacy	→	Professional mitigation	0.337	0.061	0.393	5.542	*	Supported
Responsibility	→	Professional mitigation	0.478	0.078	0.529	6.139	*	Supported
Risk perception	→	Professional mitigation	0.122	0.054	0.102	2.279	0.023	Supported
Hypothetical distance	→	Professional mitigation	0.147	0.049	0.182	2.298	0.003	Supported

*Sig* significance

*p < 0.001

The variance explanatory power (R2) value in conceptual model 2 is 90% for the intention to mitigation professional behaviors in agriculture. The study model predicts 78%, 13%, 49%, and 61% of variance changes in hypothetical distance, risk perception, personal efficacy, and responsibility, respectively.

Belief in the occurrence climate change is the strongest predictor of the intention to implement personal mitigation behaviors (β = 0.411, *t* = 5.374, *p* < 0.0001), while responsibility is the strongest predictor of the intention to engage in professional mitigation behaviors (β = 0.529, *t* = 6.139, *p* < 0.0001).

## Discussion

Globally, just as in past scientific research and political debates, climate change mitigation activities are attracting much attention both in public debate and academic research. This study uses a conceptual framework to predict the personal (Model 1) and professional (Model 2) behavioral intentions of agricultural experts to take action to reduce GHG emissions. The results show that both models explain high and very high percentages of the intention to implement both personal and professional mitigation behaviors. According to the findings, hypothetical distance is significantly associated with intention to reduce GHG emission, such that more belief in climate change happening is consistent with a greater intention to mitigate. People are more likely to engage in personal and professional mitigating behaviors if they are less skeptical and more confident about the occurrence of climate change and, in fact, know their hypothetical distance to climate change is low. Therefore, the findings emphasize the importance of reducing psychological distance, and specifically hypothetical distance, to reduce GHG emissions. This reduction of psychological distance probably leads to increased anxiety and, in turn, an increased tendency to act. The findings also show that perceptions of risks in the next 25 years, which can also be defined as concerns, directly affect the intention to implement personal and professional mitigation behaviors. Therefore, the more people feel that climate change in the next 25 years will affect human health and economic status, both globally and within Iran, the more they intend to implement mitigation behaviors. In this regard, Weber et al. (2010) highlight that if individuals do not suppose that climate change is occurring or do not perceive climate change as a threat to their livelihood, then it is more likely they will not take action to mitigate climate change. Spence et al. (2012) show that concerns about the effects of climate change are associated with intention to act. Arbuckle et al. (2013) consider the vulnerability perceived by farmers to be important and believe that farmers’ concerns about the impact of climate change are key to successful adaptation and mitigation of the effects of climate change. Yazdanpanah et al. (2022b) find that farmers’ risk perception at the farm scale plays a mediating role between overall climate belief and mitigation practices. However, there are different findings about the effect of belief in climate change happening and intention. For example, O’Connor et al. (2002) find no association between believing in a rise in temperature and supporting politics or expressing the possibility of participating in voluntary GHG mitigation behaviors.

Findings confirm that personal efficacy directly influences mitigation intention. People are more interested in implementing mitigation actions if they believe that their individual responses in daily life and in agricultural education will reduce GHG emissions. In this regard, Gifford (2011) acknowledges ‘limited cognition,’ which demonstrates itself as low self-efficacy or inefficacy as one of seven “dragons of inaction”. Inefficacy views can arise from the insight that climate variability is an unavoidable and, consequently, that individual behaviors, or even the mitigation efforts of a solitary cluster or country, will have a minimal outcome.

Risk salience, which refers to the proximity and experience of the adverse effects of climate change, influences belief in climate change happening or hypothetical distance. Recent personal experiences also strongly affect risk perception. Although personal experience of the serious consequences of global warming is still rare in many parts of the world, its effects are highly visible on agricultural land and in other vulnerable rural areas. For example, observing people or crops and lands that have been damaged by climate change leads to understanding of the dangers of climate change. Further, Carlton and Jacobson (2013) conclude that risk salience is one of the important factors in determining the perception of risk. The notion that the risk salience of climate change provides a potentially important path to intention is widely confirmed. Demski et al. (2017) show that personal issue salience directly affects behavioral intention and support for mitigation policies. In addition, Broomell et al. (2015) study of 11,000 respondents from 24 countries finds that personal experience with global warming corresponds to intention to take specific measures, like using less air conditioning in the summer. Such personal experiences may lead to greater familiarity with risk and, thus, to greater understanding by individuals (Demski et al. (2017)).

Evidence supports that agricultural experts’ views on personal responsibility are generally positive and significant in influencing mitigation. This finding indicates the importance of personal responsibility in influencing mitigation behavior. In terms of responsibility, personal responsibility refers to agricultural experts’ beliefs about doing something for a better future and refers all human responsible for reducing GHG emissions. Therefore, individuals are more likely to engage in mitigating behaviors if they know humans are responsible for the causes of climate change and have high risk perception.

There are several mediators between risk experience, risk salience, institutional trust, and the intention to engage in mitigation behaviors. It is important to increase risk salience or provide opportunities for direct experience among agricultural professionals. For this purpose, it is possible to increase the practical visits by agricultural experts to farms where crops have been damaged due to climate change, as well as visits with farmers whose health has been threatened. Indeed, direct observation of farms and crops damaged is necessary to facilitate real communication and increased personal observation of damaged crops by agricultural experts and researchers. Viewing products damaged by extreme heat or water shortages, thus gaining objective and personal experience with climate change events, can have three important consequences: First, it increases risk salience; second, it increases risk perception; and, third, it reduces the hypothetical distance, thus strengthening people’s beliefs about the reality of climate change. The most successful way to strengthen mitigation involves government interventions in agricultural service organizations that focus on local, tangible, and practical aspects. Executive policies should draw office-based experts to field experiences on agricultural land, thus increasing the likelihood of implementing mitigation strategies both personally and professionally.

Institutional trust plays an important role in believing in climate change and personal efficacy. Trust is especially important when the level of uncertainty is high and the level of knowledge is low, such as those for climate change risks. In these situations, people depend on information provided by risk managers, seeking their knowledge before making informed decisions. The more experts feel that complete, up-to-date, and accurate information is being provided by government agencies, the more they believe in climate change. In a meta-analysis, Cologna and Siegrist (2020) show that trusting that institutions provide relevant information is associated with climate-friendly behaviors, but this relationship is weak. Agricultural experts who believe that information from the government and other upstream organizations is accurate, up-to-date, and timely will also have more confidence in their ability to take action to mitigate climate change. The findings also show that the NEP significantly affects beliefs in climate change and personnel efficacy. This construct has an indirect effect on the intention to mitigate both personal and professional behaviors. In this regard, Bouman et al. (2020) show that stronger endorsements of biospheric values are coupled with greater commitment to weather mitigation behaviors.

The explanatory power of the model is 68% for personal behavioral intention and 90% for professional behavioral intention. Explanatory power of endogenous latent variables exceeds the recommended value of 0.5 (Yu et al. 2019) for both personal and professional behaviors, which shows that the model is strong and stable. However, the power of explanation in professional intentions is much higher than personal behavioral intention. In other word, occupational mitigation behaviors are more influenced by perceptions. Personal behaviors are probably perceived as being more costly than professional behaviors, thus economic factors also influence implementation of diminishing personal behaviors. The better predictive power of the professional model reveals there may be more factors that influence personal mitigation behaviors beyond those captured in our study.

From a theoretical perspective, the findings from this study represent an important step in developing a comprehensive understanding of the mitigation behaviors of Iranian agricultural experts, which can be used to adjust their communication and education systems in response to climate change.

Policy interventions should emphasize growing community and expert efforts in agricultural extension for actual mitigation performances and eliminating obstacles. In this regard, there is a clear need to improve communication efforts that emphasize and reveal the efficiency of individual actions and to create a stronger sense of obligation for addressing climate change. Public education must clearly address misunderstandings and identify those actions that are most effective in mitigating climate change. In this regard, sources of information, including, among others, the government, must be understood as trusted information sources.

Considering that the strongest predictor of the intention to implement personal actions is hypothetical distance and the strongest predictor of professional intentions is responsibility, our clearest suggestion is to focus on these two concepts. In order to reduce the hypothetical distance, it is suggested that agricultural experts should be frequently exposed to general and detailed information through climate change reports and statistics (temperature, precipitation,…) in different ways. These can include, among others, radio, television, and other mass media that increase awareness about this phenomenon and the need to respond to it. Thus, they will increase mitigation measures in their daily lives. In order to improve responsibility, it is necessary to learn about the causes of climate change, the role of human activities, and the role of the agricultural sector, especially in the workplace.

Since climate risk perception is a precondition for effective climate communication and mitigation, agricultural politicians and organizations need to increase the discourses on climate risks through the media, farmers’ associations, and other farmer groups. Improving agricultural specialists’ knowledge and opinions of risk issues with respect to climate change could be one long-term structural reply to address climate variability.

## Conclusions and Limitations

People who think climate change is hypothetical, who do not recognize the behaviors that emit GHG and its consequences, ignore their responsibility for climate change, or have a low perceived risk, are unlikely to support GHG mitigation strategies or modify their behavior to reduce GHG emissions. Individuals may be anxious about climate change and want to do the right thing, but they are unlikely to do the right thing if they do not know that their behaviors – like using cars or consumption that results in trees being cut down – are directly linked to climate change. Therefore, accurate understanding of the causes of climate change can help improve the accountability and effectiveness of perceived responses and help provide a strong cognitive predictor of GHG mitigation measures. This contributes to current debates about the role of psychological distance and skepticism about climate change as a potential barrier to both public and professional participation in mitigation. Researchers should equally consider the possible limitations of climate change approximation and recognize that bringing the effects of climate change closer is unlikely to amplify climate change mitigation alone. Instead changing psychological distance should be associated with perceived risk, increased efficacy, and responsibility. Therefore, educating people about environmental issues needs to cover different dimensions if climate change mitigation behavior is to be induced.

This study contributes to the literature by (1) empirically examining the moderating role of risk perception, hypothetical distance, personal effectiveness, and responsibility between institutional trust, risk salience, and the new ecological paradigm with intention to implement personal and professional mitigation; and (2) developing a conceptual model that combines VBN and NAM with hypothetical distances. This research has several limits that must be considered when interpreting the outcomes. First, the analysis in this study uses non-experimental and cross-sectional data. Future research should duplicate this study using longitudinal data in which samples are randomly separated into groups. Second, the random sample used in this study is from Khuzestan province, Iran, where people’s livelihood is highly dependent on agriculture. Therefore, the generalizability of our result is limited to this province only. Future research should include examples from other parts of Iran and other developing countries. In addition, this study focuses on the intentions of individuals, although, in many cases, individuals fail to translate intention into behavior, so future studies should examine the actual behavior of individuals. Other groups, like farmers and policymakers, among others, may have different values, perceptions, and behaviors toward mitigating climate change; thus, their views should be examined. The lack of participation in mitigative measures can be due to cognitive constraints: this must be considered in future work. Hence, future research should also consider the effect of other social psychological constructs like norms, awareness, social trust, perceived barriers, perceived costs, and cultural factors on mitigating behaviors in response to climate change.

## Data Availability

Some or all data, models, or code that support the findings of this study are available from the corresponding author upon reasonable request.
